# Ingroup preferences, segregation, and intergroup contact in neighborhoods and civic organizations

**DOI:** 10.1093/pnasnexus/pgaf256

**Published:** 2025-09-02

**Authors:** Kasimir Dederichs, Rob Franken, Dingeman Wiertz, Jochem Tolsma

**Affiliations:** Nuffield College, University of Oxford, New Road, Oxford OX1 1NF, United Kingdom; Department of Sociology, Utrecht University, Padualaan 14, 3584 CH Utrecht, the Netherlands; UCL Social Research Institute, University College London, 55-59 Gordon Square, London WC1H 0NU, United Kingdom; Department of Sociology, Radboud University Nijmegen, Thomas van Aquinostraat 4, 6525 GD Nijmegen, the Netherlands; Department of Sociology, University of Groningen, Grote Kruisstraat 2/1, 9712 TS Groningen, the Netherlands

**Keywords:** ingroup preferences, segregation, neighborhoods, civic organizations, conjoint experiments

## Abstract

Segregation perpetuates social inequalities and undermines social cohesion. It can already emerge if individuals act upon weak preferences to associate with similar others. Yet, little remains known about how such ingroup preferences compare across social settings and different identity dimensions. To address this gap and to isolate ingroup preferences from other drivers of segregation, three large-scale, preregistered conjoint experiments on choices of neighborhoods and civic organizations were conducted (*N*_1_ = 2,733, *N*_2_ = 2,743, *N*_3_ = 2,707). The results reveal powerful ingroup preferences in both settings and across all studied dimensions (age, ethnicity, education). These preferences are strongest among individuals with little real-life exposure to outgroups and do not depend on the expected intensity of contact. As an exception, lower-educated individuals display no ingroup preferences along educational lines. Altogether, the results highlight that ingroup preferences are pervasive, can pose a critical obstacle to intergroup contact, and should thus be carefully considered in desegregation efforts.

Significance Statement This study examines to what extent individuals prefer social settings where other people have similar sociodemographic characteristics as themselves. We compare such ingroup preferences regarding the composition of neighborhoods and civic organizations with respect to age, education, and ethnicity. Three survey experiments show that ingroup preferences (i) are commonplace along all three dimensions and in both settings; (ii) are stronger among individuals who have little exposure to outgroups; (iii) but do not depend on the expected intensity of contact. Lower-educated individuals are the only exception to this pattern, as they do not display any ingroup preferences along educational lines. Overall, these findings underscore the pervasiveness of ingroup preferences and their relevance for any efforts to reduce segregation.

## Introduction

Many people live their lives in social bubbles—segregated settings that primarily offer opportunities for contact with others similar to oneself. This holds true whether we consider workplaces, schools, neighborhoods, civic organizations, online networks, or other contexts. As a result, social connections bridging across group boundaries remain rare, even though societies at large are becoming more diverse (1–5). This lack of intergroup contact can have grave consequences, perpetuating social inequalities and undermining social cohesion (6–8). In this context, interventions in confined settings such as sports leagues and military units have shown that intergroup interactions can help overcome prejudice and reduce social distance between groups, among other beneficial effects (9–15). In everyday life, however, such interactions often do not materialize due to widespread segregation. Therefore, and notwithstanding that intergroup contact can also have negative effects (16–20), segregation represents a critical obstacle to an inclusive and cohesive society.

One key explanation for the existence of segregation is that individuals prefer to interact with people similar to themselves—a phenomenon also referred to as choice homophily. Ingroup preferences may reflect a genuine desire for sociodemographic similarity, or derive from shared interests, tastes, and opinions among individuals with the same sociodemographic characteristics (21). Even if individuals act upon weak ingroup preferences, strong segregation can be the aggregate result, given that contacts with ingroup members beget more ingroup contacts through triadic closure (i.e. two individuals connected through a third person forming a direct connection) and peer influences on the selection of new interaction settings (22–26). Since the resulting segregation limits opportunities for intergroup contact, stereotypical beliefs about outgroups and preferences to avoid intergroup contact often remain unchallenged. Accordingly, a vicious cycle may arise in which limited intergroup contact consolidates ingroup preferences, which in turn steer people into settings with limited potential for contact with outgroups.

Despite these potentially far-reaching consequences of ingroup preferences, we know little about their actual salience for social sorting processes in everyday life. While existing research does examine ingroup preferences along various social dimensions (e.g. ethnicity, gender, partisanship) in various settings (e.g. neighborhoods, schools, workplaces) (27–37), this research is subject to three important limitations. First, previous studies only consider one social setting and one social dimension at a time. This implies that the strength of ingroup preferences cannot be compared across settings or different identity dimensions. Moreover, it remains unclear whether ingroup preferences along one dimension (e.g. ethnicity) manifest themselves independently or as a by-product of ingroup preferences along another dimension (e.g. education), as many people might associate certain social characteristics with other characteristics (e.g. presuming that ethnic minority members are lower-educated). Second, previous studies do not examine whether ingroup preferences vary by individuals' exposure to outgroups, leaving untested a critical precondition for the emergence of vicious “exposure-preference” cycles as described above. Finally, previous research has not considered whether the strength of ingroup preferences depends on the expected intensity of contact. This is problematic because, whereas closer contacts are argued to matter more for improving intergroup relations than more superficial exposures do (6), ingroup preferences may well become stronger when the expected intensity of contact increases (38).

This study addresses these limitations, providing a rigorous evaluation of the strength of ingroup preferences across multiple domains of everyday life. Considering ingroup preferences in terms of age, ethnicity, and education level, we systematically compare how ingroup preferences vary across different identity dimensions and between different subgroups (e.g. those with and without a college degree). Furthermore, we compare the salience of ingroup preferences between two different settings: neighborhoods and civic organizations (e.g. sports clubs, cultural associations). Both are widely considered core pillars of community cohesion (39, 40), albeit in different ways: neighborhoods structure residents' long-term opportunities for social interaction (41, 42), but neighbors do not necessarily share common interests that would directly promote the formation of social ties among them. Civic organizations, by contrast, typically do bring people together who share similar interests (e.g. in a certain sport or cultural practice), are often explicitly organized around social interactions and cooperation, and can play a key role in the grassroots-level coordination of interests (43). Although there are typically lower entry barriers to civic organizations vis-à-vis neighborhoods, both settings are in practice often strongly segregated along multiple sociodemographic dimensions (44–51). By including both settings in our analysis, we provide a more comprehensive picture of how ingroup preferences undermine intergroup contact in communities than previous studies that have only looked at neighborhoods (no previous study has analyzed ingroup preferences regarding civic organizations). Moreover, direct comparisons between both settings can deliver vital insights into which settings are most fruitful for stimulating intergroup contact as well as the conditions under which ingroup preferences are most potent. If, for example, ingroup preferences turn out to be stronger in one setting vis-à-vis the other, this could guide future research into the conditions that trigger or activate ingroup biases. If, however, we observe little difference between the settings, this would underscore the universality of ingroup preferences (52).

When assessing the role of ingroup preferences in producing and sustaining segregation, a major challenge is to isolate their influence from that of differences in opportunity structures. After all, segregation may also result from differences in spatial and financial constraints across groups (53), as well as discrimination by relevant gatekeepers (54, 55), all of which limit the extent to which individuals can act upon their preferences. The resulting variation in choice options across social groups can, however, not easily be adjusted for in analyses based on observational data, which usually only includes information on those settings individuals end up in, providing no insight into which alternatives they may have considered. Therefore, the influences of preferences and opportunities cannot be straightforwardly teased apart using observational data. Likewise, one cannot identify ingroup preferences by directly asking individuals about their motives for choosing particular settings, as such questions would likely elicit social desirability bias, post hoc rationalizations, or other response biases (56–59).

To circumvent these problems, this study relies on three preregistered conjoint experiments (60, 61), in which participants repeatedly had to choose between paired profiles of neighborhoods or civic organizations with varying social compositions. Since the experimental design randomized the choice sets individuals were exposed to, their preferences regarding social similarity can be isolated from any structural drivers of segregation. In other words, differences in opportunity structures were experimentally controlled for. Furthermore, the neighborhood and organization profiles differed along various dimensions other than their social composition alone (e.g. financial costs, vicinity, social cohesion). This multifaceted setup reduces the risk that individuals display socially desirable response behaviors instead of revealing their true preferences regarding social similarity (60, 62). Moreover, it enables comparisons between the influence of the social composition and other characteristics of the settings.

In contrast to the common reliance of experimental studies on convenience samples, our conjoint experiments were embedded in two large-scale, nationally representative Dutch panel studies with high response rates. Experiments 1 and 2, about choices between neighborhoods and civic organizations, were conducted through the Longitudinal Internet studies for the Social Sciences (LISS) panel. Among the same 2,750 respondents, 2,733 completed experiment 1 and 2,743 completed experiment 2, resulting in 21,875 choice tasks overall. In this data collection, we additionally gathered detailed information on the social composition of the neighborhoods and organizations that these respondents were part of in real life. This enables us to explore how ingroup preferences are linked to real-life segregation. Experiment 3 was conducted through the Transitions into Active Living (TRIAL) panel among 2,707 respondents who completed 8,121 choice tasks in which they had to choose between sports clubs. Sports clubs are not only the most popular type of civic organization in the Netherlands and most other European countries (63), they are usually also subdivided into smaller units (e.g. teams, training groups) that shape opportunities for interaction. This structure enables us to examine whether people find it more important to be surrounded by similar others when interactions are expected to be more intense.

Our experiments demonstrate the prevalence of strong and remarkably consistent ingroup preferences when people choose neighborhoods and civic organizations. These preferences manifest themselves independently across all studied social dimensions and for most subgroups, with realistic increases in outgroup shares undermining the appeal of settings to a similar degree as realistic but substantial increases in travel time (to local amenities or civic organization). Furthermore, individuals who face less exposure to outgroups in real life tend to display stronger ingroup preferences, underscoring the possible threat of vicious cycles whereby ingroup exposure and ingroup preferences reinforce each other. Finally, whereas ingroup preferences do not strengthen when the expected intensity of contact increases, their parallel existence at multiple levels (e.g. regarding sports clubs and teams within them) implies that segregation is likely to increase at each sorting node. One exception to these otherwise consistent patterns is that, in contrast to individuals with a college degree, those without one exhibit no ingroup preferences along educational lines. Accordingly, segregation by education level is more strongly shaped by the preferences of the college-educated than by those of the less-educated. By offering systematic evidence on the pervasiveness of ingroup preferences across different social settings and identity dimensions, this study helps to better understand patterns of segregation in everyday life, with implications for how to reduce segregation and foster social cohesion.

## Results

### Segregation in neighborhoods and civic organizations

**Fig. 1.**
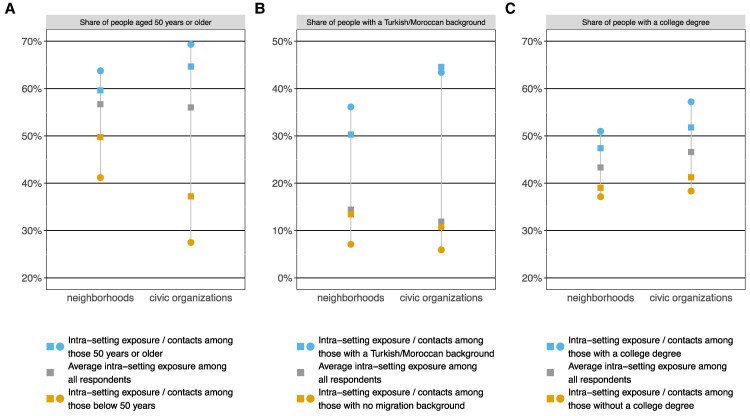
Segregation across and within neighborhoods and civic organizations. Note: Squares represent the composition of a given setting (survey question: “Now think about everyone in your [neighborhood/organization]. How many of these people… Are 50 years or older? Have a Turkish or Moroccan background? Have a college degree?). Circles represent the composition of individuals' networks of regular contacts within a setting (survey question: “Now think about everyone with whom you regularly interact in your [neighborhood/organization]. How many of these people…). Both questions were answered using a slider from 0 to 100% (or a “don’t know” button). Respondents involved in multiple civic organizations were instructed to think about the organization they spent most time on. This figure is based on the responses given by 2,620 respondents; see Table S1 for more details about their sociodemographic characteristics.

Figure 1 provides the background for the conjoint experiments, illustrating the prevalence of segregation in Dutch neighborhoods and civic organizations in terms of *exposure to* and *contact with* different social groups, as estimated by the respondents themselves. Sports clubs were by far the most common type of civic organization, followed by cultural and religious associations (see Table S1 for an overview of all types of civic organizations in our data). The gray squares depict the average presence of individuals aged over 50 years (see Panel A), with a Turkish or Moroccan background (the largest ethnic minorities, see Panel B), or a college degree (see Panel C) in individuals' current neighborhoods and organizations. The blue and orange squares separate these average exposures by individuals' own characteristics, plotting quantities referred to as the isolation and interaction index (64). The deviations of the blue squares at the top and the orange squares at the bottom from the gray squares in the middle signify clear segregation *across* neighborhoods and civic organizations. For example, the share of neighbors with a college degree is 39% among individuals *without* a college degree vis-à-vis 47% among those *with* such a degree (see Fig. 1C). In general, segregation across civic organizations is stronger than across neighborhoods, as indicated by wider gaps between the blue and orange squares. The blue circles at the top and the orange circles at the bottom depict the average composition of individuals' interaction networks within their neighborhoods and organizations. These circles are even further apart from each other than the associated squares (in all but one case). This demonstrates that intergroup contact is not only impeded by segregation across neighborhoods and organizations but also by segregation *within* these settings. Overall, Fig. 1 underlines the pervasiveness of segregation in social life, illustrating its prevalence across multiple settings, multiple identity dimensions, and both across and within settings. We next turn to ingroup preferences as a potential driver of this segregation.

### Ingroup preferences in choosing neighborhoods and civic organizations

In experiments 1 and 2, the same respondents were repeatedly shown paired profiles of fictive neighborhoods and civic organizations they could move to or join. For each profile pair, they had to indicate which alternative they would prefer. All neighborhood and organization profiles varied in terms of their age, ethnic, and educational composition, as well as their costs (rent/membership fee), travel time (to amenities/from home), social cohesion (how well neighbors/members know each other), and respondents' preexisting ties to people inside the neighborhood or organization (see Table S2 for the exact phrasing and potential values of all profile attributes). The profiles shown to respondents were randomized. As a consequence of our sampling design, all respondents were active in at least one civic organization in real life, ensuring they were familiar with making choices about civic affiliations (see Table S1 for a descriptive overview of the sample).

**Fig. 2.**
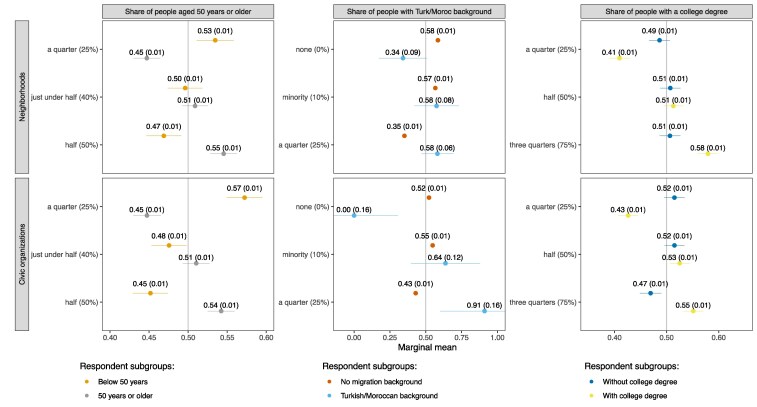
Marginal means of neighborhoods' and organizations' social composition attributes by respondents' age, ethnicity, and education level (experiments 1 and 2). Note: Marginal means reflect the average probability with which a profile with a particular attribute is chosen. SEs are displayed in parentheses, and the error bars represent 95% CIs. All estimates have been corrected for measurement error following Clayton et al. (65). The instructions for each choice task read: “Imagine you leave your current [neighborhood/civic organization] and are looking for a new one. Which one would you choose?” The civic organizations which respondents had to choose between were of the same type as their current (most time-consuming) organization. This figure is based on the choices made by 2,750 respondents, out of whom 2,733 participated in the experiment on neighborhoods and 2,743 in the experiment on civic organizations. Each respondent was asked to complete four choice tasks for each setting. Figure S1 shows the marginal means across the entire sample, including for the profile attributes omitted here. For further supplemental materials on this figure, see Tables S2–S4 and Fig. S2.

Figure 2 presents the marginal means of neighborhoods' and organizations' social composition attributes by respondents' own sociodemographics. Marginal means offer an intuitive way to analyze subgroup differences in conjoint experiments by providing the average probability with which a profile with a particular attribute is chosen (66). Because each choice set contains two profiles, marginal means above 0.5 reflect preferences in favor of an attribute and those below 0.5 preferences against it.

The leftmost panels of Fig. 2 show consistent age-based ingroup preferences in both settings. While respondents under 50 prefer neighborhoods and organizations where *fewer* people are 50 years or older, respondents over 50 have markedly different preferences, preferring settings where *more* people are 50 years or older. Those under 50 display slightly stronger ingroup preferences when choosing civic organizations vis-à-vis neighborhoods, as indicated by statistically significant cross-setting differences in marginal means. No such differences are found among respondents over 50. See Table S3 for more detailed results of statistical tests of any cross-setting differences shown in Fig. 2.

The middle panels of Fig. 2 also demonstrate significant ethnic ingroup preferences regarding neighborhoods and civic organizations, although the exact patterns differ by respondents' own background. Those without a migration background are largely indifferent between settings with 0 and 10% minority shares but avoid settings with larger minority shares. This tendency is stronger for residential vis-à-vis organizational choices. By contrast, among respondents with a Turkish or Moroccan background, ethnic ingroup preferences are especially strong for organizational choices, although the modest underlying sample size calls for caution in the interpretation of this result (56 respondents had a Turkish or Moroccan background).

The rightmost panels of Fig. 2 finally reveal strong education-based ingroup preferences among respondents with a college degree, which are slightly more pronounced in the neighborhood vis-à-vis organizational setting. Strikingly, however, those without a college degree are largely indifferent between neighborhoods and organizations with different educational compositions, displaying only a weak preference against civic organizations with 75% college-educated members. This may reflect that education is a weaker source of identity among those without a college degree (67, 68) or that this group is affected by countervailing forces that offset their ingroup preferences. For example, those without a college degree might still appreciate settings with many similarly educated individuals but have status-seeking motives (69), or perceptions that settings with more college-educated people function better or have other desirable features (e.g. more highly educated neighborhoods having higher-quality schools, better-maintained infrastructure, and lower crime rates). Equally, whereas higher-educated individuals typically report lower levels of prejudice (70), our results may indicate that such tolerance primarily constitutes lip service but does not really find expression in people's everyday lives.

In sum, Fig. 2 demonstrates the pervasiveness of ingroup preferences in neighborhood and organizational choices: barring the absence of strong education-based ingroup preferences among those without a college degree, individuals consistently prefer settings where they can expect to encounter more people of similar age, ethnicity, and education as themselves. These preferences manifest themselves for each social dimension independently of the other considered dimensions. For all age- and education-based subgroups, the strength of ingroup preferences is also remarkably similar across the neighborhood and organizational settings. Regarding ethnicity, however, we observe notable differences: Individuals without a migration background have stronger ethnic ingroup preferences concerning neighborhoods, whereas those with a Turkish or Moroccan background seem to have stronger ethnic ingroup preferences concerning civic organizations. Moreover, the ethnic ingroup preferences of individuals without a migration background seem to follow a threshold function. These findings are important for future research seeking to model segregation.

To further illustrate the strength of the ingroup preferences, we have benchmarked them in Table 1 against respondents' preferences for settings involving less travel time. More specifically, we have calculated, for each sociodemographic subgroup, which changes in travel time yield identical differences in marginal means as particular changes in social composition. We interpret these quantities as the number of additional minutes individuals are willing to travel for settings with more preferable social compositions. Table 1 shows, for example, that individuals under 50 years are on average willing to travel ∼5 min longer for a civic organization where 25 as opposed to 50% of members are over 50. Individuals without a migration background are on average willing to accept close to 10-min additional travel time to amenities if this means they can live in a neighborhood with 0 as opposed to 25% residents with a Turkish or Moroccan background. Individuals with a college degree are willing to incur a travel time penalty of more than 6 min to live in a neighborhood where 75 as opposed to 25% of their neighbors are college-educated as well. These figures, which increase if we look at more selective subgroups (e.g. those under 35 years rather than those under 50 years), underscore the significant value individuals attach to social similarity—especially considering that all travel times in our experiments fall between 10 and 20 min (a realistic range for the Netherlands as a small, densely populated country) and that they refer to journeys that typically recur multiple times per week. Table S4 provides the compensatory travel times for all subgroups and composition-level comparisons.

**Table 1.** Benchmarking ingroup preferences against preferences for reduced travel time (experiments 1 and 2).

Setting	Focal subgroup	Change in social composition	Compensatory travel time for change in social composition (in min)
Neighborhoods	Below 50 years	25 → 50% 50 years or older	−2.9
	50 years or older	25 → 50% 50 years or older	3.9
	No migration background	0 → 25% Turkish or Moroccan background	9.8
	Turkish or Moroccan background	0 → 25% Turkish or Moroccan background	15.8
	Without college degree	25 → 75% With college degree	1.0
	With college degree	25 → 75% With college degree	6.4
Organizations	Below 50 years	25 → 50% 50 years or older	−4.9
	50 years or older	25 → 50% 50 years or older	5.5
	No migration background	0 → 25% Turkish or Moroccan background	−4.7
	Turkish or Moroccan background	0 → 25% Turkish or Moroccan background	55.2
	Without college degree	25 → 75% With college degree	−2.4
	With college degree	25 → 75% With college degree	6.1

Note: For a full overview across all subgroups and all possible changes in social composition, see Table S2e.

### Ingroup preferences and real-life segregation

**Fig. 3.**
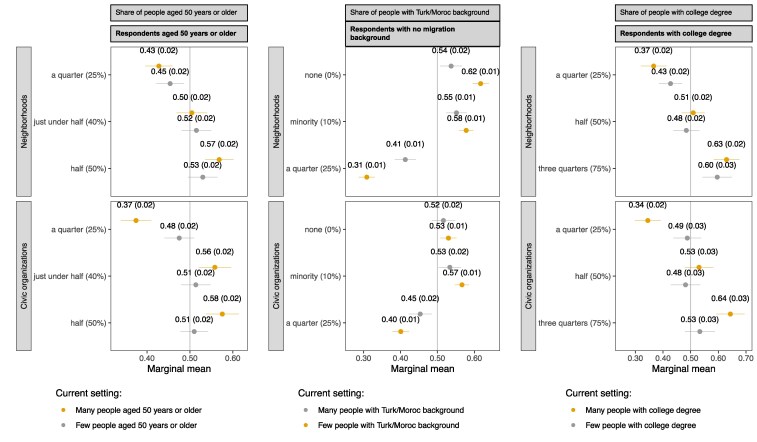
Marginal means of neighborhoods' and organizations' social composition attributes by the composition of respondents' current neighborhoods and organizations, for selected sociodemographic groups (experiments 1 and 2). Note: Marginal means reflect the average probability with which a profile with a particular attribute is chosen. SEs are displayed in parentheses, and the error bars represent 95% CIs. All estimates have been corrected for measurement error following Clayton et al. (65). See the notes to Fig. 2 for the instructions provided to respondents. The composition of respondents' current neighborhoods and organizations was estimated by the respondents; see the notes to Fig. 1 for the associated survey questions. This figure is based on a smaller number of respondents than Fig. 2 (*n* = 2,523 instead of 2,750 unique respondents) because only respondents belonging to the three subgroups are considered and because respondents who reported not to know the composition of their setting were excluded. Respondents were asked to complete four choice sets per setting. Neighborhoods and organizations with “many” (“few”) people aged 50 years or older/with a college degree are those in the highest (lowest) quartile of the composition distributions of respondents' current settings, within each sociodemographic subsample. Neighborhoods and organizations with “many” (“few”) people with a Turkish/Moroccan background are those above (below) the mean of the composition distributions of respondents' current settings, among respondents without a migration background. Thus, orange (gray) dots refer to respondents with few (many) outgroup members in their current setting. For further supplemental materials on this figure, see Figs. S3 and S4.

To explore how ingroup preferences are linked to real-life segregation, Fig. 3 shows how these preferences vary depending on the composition of respondents' current neighborhoods and organizations (as estimated by the respondents themselves). Presenting marginal means and focusing on one sociodemographic subgroup per dimension, this figure demonstrates that individuals who have in real life less exposure to outgroups tend to display stronger ingroup preferences. That is, the gradients between the orange points in Fig. 3 (corresponding to individuals embedded in settings with low outgroup exposure) generally tend to be steeper than those between the gray points (corresponding to individuals embedded in settings with high outgroup exposure). In other words, within each panel, the orange points tend to be located further away from 0.5 than the gray ones.

As an illustration, the top-middle panel shows, among individuals without a migration background, a stronger aversion to neighborhoods with many residents of Turkish or Moroccan descent for those living in neighborhoods with fewer such residents: among residents of neighborhoods with relatively *few* neighbors of Turkish or Moroccan background, the marginal means are, respectively, 0.62 and 0.31 for neighborhoods where 0 and 25% of residents have such backgrounds. Among residents of neighborhoods with relatively *many* neighbors of Turkish of Moroccan background, the respective marginal means are 0.54 and 0.41. In other words, residents of neighborhoods with few neighbors of Turkish or Moroccan background have—compared with their counterparts residing in neighborhoods with many neighbors of such backgrounds—a stronger aversion against neighbors of Turkish or Moroccan background. Likewise, regarding organizational choices, the bottom-left and bottom-right panels show virtually no ingroup preferences for individuals embedded in organizations with relatively high exposure to age-based and educational outgroups but strong ingroup preferences for those embedded in organizations with limited exposure to outgroups. Similar links between individuals' exposure to outgroups and their ingroup preferences can also be observed for the sociodemographic subgroups omitted from Fig. 3; see Fig. S3. In sum, individuals whose current settings contain more ingroup members and thus more closely resemble social bubbles tend to hold stronger ingroup preferences than their counterparts embedded in more diverse settings.

The observed link between the strength of ingroup preferences and current outgroup exposure could signify either that individuals with stronger ingroup preferences are more likely to end up in ingroup-dominated settings as a result of these preferences (i.e. ingroup preferences leading to segregation), or that they develop stronger ingroup preferences in response to being situated in such settings (i.e. segregation shaping ingroup preferences). While our empirical analysis cannot distinguish between these scenarios, both concern theoretical pathways through which ingroup preferences may contribute to segregation: they could directly drive social sorting processes or make any preexisting sorting more enduring. As such, the results in Fig. 3 substantiate the plausibility of vicious cycles whereby ingroup preferences and limited outgroup exposure reinforce each other.

### Ingroup preferences as expected contact intensifies

**Fig. 4.**
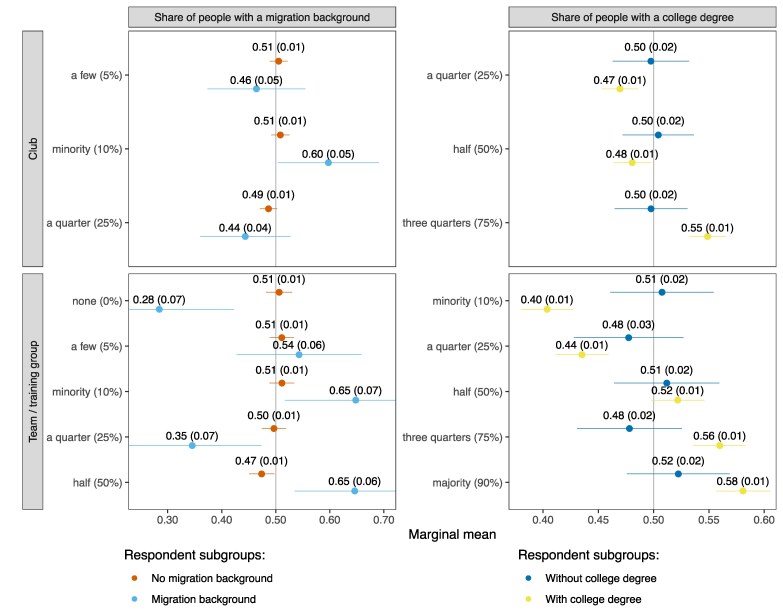
Marginal means of clubs' and teams'/training groups' social composition attributes by respondents' migration background and education level (experiment 3). Note: Marginal means reflect the average probability with which a profile including a particular attribute is chosen. SEs are displayed in parentheses, and the error bars represent 95% CIs. All estimates have been corrected for measurement error following Clayton et al. (65). The instructions to experiment 3 read: “Imagine you are looking for a new sports club, for example because you moved home. Imagine you can choose between the following two sports clubs. Which one would you choose?”. This figure is based on the choices made by 2,707 respondents. Each respondent was asked to complete three choice tasks. Figure S5 shows the marginal means across the entire sample, including for the profile attributes omitted here. For further supplemental materials on this figure, see Tables S5–S6 and Fig. S6.

To address whether ingroup preferences are stronger when contact is expected to be more likely or intense, we conducted experiment 3, where 2,707 respondents repeatedly had to choose between pairs of sports clubs, for a total of 8,121 choice tasks. For each club, they received information about the composition of the entire club and the team/training group they would join. The underlying idea is that sports participants generally interact more often and more intimately with members from their team/training group than with other club members. Regarding the composition dimensions, this experiment only considered ethnicity—phrased in terms of having “a migration background”—and education level, because sports groups are often age-segregated by design. The other profile attributes are travel time from home, club ethos (socially versus performance-oriented), preexisting ties to club members, and meeting frequency (see Table S6 for the exact phrasing and potential values of all profile attributes and Table S5 for a descriptive overview of the sample). Figure 4 shows the marginal means of the social composition attributes by respondents' own migration background and education level.

Focusing first on education level, the top-right panel of Fig. 4 shows education-based ingroup preferences at the club level that are consistent with the results of experiment 2: college-educated respondents display education-based ingroup preferences, but those without a college degree do not, possibly due to the aforementioned counter-mechanisms less-educated individuals may be subject to. The bottom-right panel reveals similar patterns at the team/training group level. Indeed, while we can here also pick up preferences toward settings with more extreme compositions (i.e. 10 or 90% college-educated), the gradients between the marginal means for the other composition categories largely match the corresponding gradients in the top-right panel. In other words, education-based ingroup preferences do not intensify when the expected amount of contact increases. Instead, individuals seem to value social similarity at the club and team/training group level to a similar degree. This might, for example, reflect that they feel more “at home” in clubs where also comembers whom they less frequently interact with share the same characteristics. In any case, the independent existence of ingroup preferences at multiple levels implies that they will add up when individuals can sequentially sort themselves at different levels. Thereby, these results offer an explanation for why in Fig. 1 the levels of segregation across individuals' contact networks in neighborhoods and organizations exceed the general levels of segregation across neighborhoods and organizations.

Regarding migration background, the two left panels of Fig. 4 also show similar preference patterns at the club and team/training group level. However, in contrast to the strong ethnic ingroup preferences displayed in Fig. 2, these panels reveal no meaningful ingroup preferences based on migration background. Having ruled out various potential reasons for these divergent results (e.g. they do not reflect that sports clubs are different from other civic organizations, or that experiment 3 is based on a sample also comprising individuals who are not involved in civic organizations), two explanations seem most plausible: first, as experiment 3 is administered to a younger sample of respondents (aged 16 to 40 years), it is possible that this specific combination of subgroup and setting (sports clubs) is associated with greater tolerance toward ethnic outgroups. This conjecture is supported by Fig. S6, where the sample of experiment 2 is restricted to the same age range as experiment 3 and split up between members of sports clubs and other organizations. This figure mirrors Fig. 4 in showing no meaningful ethnic ingroup preferences regarding sports clubs among respondents under 40 years. Second, the composition dimension in question is phrased more broadly in experiment 3 vis-à-vis experiment 2, referring to people “with a migration background” versus those “with a Turkish or Moroccan background.” The former constitute a much more heterogeneous population, including groups that are culturally more similar to the ethnic majority and that face less discrimination than people of Turkish or Moroccan descent (e.g. migrants from neighboring European countries). Altogether, these discordant results emphasize that ingroup preferences along one dimension (in this case: migration background) may vary depending on individuals' other sociodemographic characteristics and that they can be highly sensitive to how ingroups and outgroups are defined and the extent to which this matches individuals' own perceptions and identities.

### Robustness checks

Several additional analyses were conducted to address potential concerns regarding the previously discussed results. First, the large degree of similarity in response behaviors between experiments 1 and 2 (on neighborhood and organizational choices) might arise because respondents may have developed response heuristics during their first experiment, which they subsequently also applied during the second experiment, without consciously thinking about differences between the settings. To address this concern, we repeated our analyses including only data from the first experiment respondents completed, taking advantage of the fact that the order in which they completed experiments 1 and 2 was randomized. The results of these analyses closely resemble those in Figs. 2 and 3 (see Fig. S2).

A second concern, involving the analyses summarized in Fig. 3, might be that the composition measures of respondents' current neighborhoods and organizations are based on respondents' own estimates. Such estimations constitute cognitively demanding tasks that may be subject to systematic biases (71). We therefore repeated the neighborhood analyses underlying Fig. 3, this time measuring the composition of respondents' current neighborhoods using administrative registers to which we were able to link our survey data. These analyses yield largely similar results as reported in Fig. 3 (see Fig. S4), showing that ingroup preferences tend to be stronger among respondents who live in neighborhoods where they are less exposed to outgroups.

## Discussion

Across the social sciences, studies of segregation have commonly assumed that individuals must have ingroup preferences when it comes to choosing interaction settings (26, 72, 73). Yet, the strength and scope of such preferences remain poorly understood to date. In which settings and for which social dimensions do they play a role? Are they linked to individuals' exposure to outgroups in everyday life? Do they depend on the intensity of contact? This study offers answers to these questions.

Most importantly, this study demonstrates the pervasiveness of ingroup preferences in social life as a critical obstacle to intergroup contact. With few exceptions, individuals consistently prefer settings where there are more people like themselves, whether we look at neighborhoods or civic organizations, and whether we consider their composition in terms of age, ethnicity, or education. The similar patterns observed across multiple settings and dimensions underline the role of ingroup preferences as a fundamental mechanism structuring social interactions. As such, the present study adds further credibility to earlier studies that showed the existence of ingroup preferences for individual dimensions (27, 28, 30, 32, 33, 37), by clarifying that such preferences are neither restricted to one particular setting nor merely represent a by-product of ingroup preferences regarding empirically correlated sociodemographic dimensions.

At the same time, our analysis uncovers several meaningful differences in the strength of ingroup preferences across settings and subgroups. In particular, whereas ingroup preferences among individuals without a migration background are stronger when it comes to choosing neighborhoods vis-à-vis organizations, the pattern seems reversed among individuals with a Turkish or Moroccan background. Moreover, educational ingroup preferences are strong among individuals with a college degree but virtually absent among those without such a degree—an important exception to the general pattern of widespread ingroup preferences, possibly reflecting perceptions among the lower-educated that more highly educated settings can help to enhance their status or possess other desirable features (74). In addition, whereas most ingroup preferences follow a linear pattern, individuals without a migration background seem to have nonlinear preferences regarding the presence of people with a Turkish and Moroccan background in their neighborhoods and organizations. They are indifferent between settings with small shares of these groups but firmly avoid settings where these groups are more strongly represented. Finally, younger adults do not display any ethnic ingroup preferences when choosing sports clubs but do so in the context of other civic organizations or neighborhoods, suggesting that—among this subgroup—sports clubs might constitute a more fertile ground for interethnic mixing. These findings add critical nuance to our headline conclusion that ingroup preferences are pervasive, and should be taken note of by policymakers and practitioners looking to combat segregation.

Another important finding is that ingroup preferences are typically more salient among individuals who are embedded in settings where they are less exposed to outgroups. This association may indicate that individuals with stronger ingroup preferences sort into more ingroup-dominated settings, thus directly causing segregation, or that a lack of exposure to outgroups in everyday life leads to more exclusive preferences, thereby making any preexisting segregation more enduring. Whichever of these scenarios is true, ingroup preferences can thus contribute to segregation, and in the plausible case that both processes take place, the result may be a vicious cycle where ingroup exposure and ingroup preferences mutually reinforce each other.

Our results further show that individuals' ingroup preferences are largely stable across different interaction levels (e.g. organizations and organizational subunits). Besides underlining the widespread salience of ingroup preferences—even for contexts that involve limited contact—this finding offers an explanation for why segregation is stronger when considering individuals' contact networks in their neighborhoods or organizations vis-à-vis the composition of these settings as a whole (see Fig. 1): Since individuals hold ingroup preferences at multiple interaction levels, these preferences will add up across “choice nodes.” In other words, the more choice nodes people have, the more segregation can be expected.

All of these findings have been derived from a unique study design consisting of multiple matched conjoint experiments embedded within two large-scale surveys that draw respondents from national population registers. Improving upon observational studies on the origins of segregation, these conjoint experiments allow us to isolate ingroup preferences from the influence of opportunity structures. More generally, conjoint experiments have been shown to possess relatively high levels of internal and external validity, even when complex and sensitive subjects are studied (58, 60, 62), and we have additionally adjusted our estimates for measurement error that may occur in such experiments (65). By repeating our experiments across multiple settings and by including multiple social dimensions, we were able to make direct comparisons regarding the existence and strength of ingroup preferences across different settings and identity dimensions. Such comparisons are indispensable for coming to a better understanding of the salience of ingroup preferences in social life and any segregation resulting from this.

At the same time, further research remains necessary. In particular, our study could not address to what extent the ingroup preferences revealed in our experiments drive real-world behaviors (i.e. moving to a certain neighborhood or joining a certain organization). Even if ingroup preferences are strong, this might not necessarily result in segregation if (i) members of different groups share preferences for other setting characteristics that are given more weight in the decision process (e.g. regarding neighborhoods, people may care more about low crime rates or the presence of high-quality schools) or (ii) opportunity structures restrict the extent to which people can act on their ingroup preferences (e.g. when there are only few organizations of a given type available, with little variation in social composition among them) (36). That said, our findings regarding respondents' contacts within their current neighborhoods and organizations show that segregation emerges even in contexts where opportunities for mixing are present (see Fig. 1), underlining that ingroup preferences are very likely to contribute to segregation and undermine intergroup contact. Still, detailed longitudinal data about individuals' ingroup preferences and the social settings they are embedded in remain needed to further disentangle these dynamics. Furthermore, our findings are obtained from a single country: the Netherlands. Therefore, their generalizability to other countries, including ones with different traditions of residential mixing, civic landscapes, and immigration histories, remains to be established.

From a policy perspective, our findings above all highlight why it is so difficult to address segregation and the resulting lack of intergroup contact top-down. Historically, policymakers have mainly aimed their desegregation efforts at improving opportunities for disadvantaged groups, for example through housing subsidies or designated social housing in advantaged neighborhoods (75, 76). Our results, however, suggest that attention must also be paid to the role of privileged groups, such as the ethnic majority and the college-educated, in sustaining segregation. Their avoidance of settings featuring more exposure to outgroups likely poses a major barrier to a durable reduction of segregation. Accordingly, merely increasing outgroup exposure without simultaneous investments to stimulate changes in people's ingroup preferences is unlikely to be a successful strategy.

It seems particularly important to address the conditions under which (existing) intergroup contact takes place, to facilitate its continuation and potential intensification, and to maximize the likelihood that it could durably improve intergroup attitudes. Civic organizations might, for example, introduce practices and rituals emphasizing commonalities and promoting identities shared by all members regardless of their sociodemographic characteristics (11, 77). In the neighborhood context, the provision of high-quality inclusive public infrastructure (e.g. playgrounds, outdoor furniture, community cafés) may help to provide the conditions for durable intergroup contact that could bring about more inclusive preferences (39). In any case, although ingroup preferences are difficult to change, it is worth developing effective approaches to this, as these preferences will inevitably be of essential importance for the ultimate success of any intervention to reduce segregation.

## Materials and methods

### Data collection and ethical compliance

Our analysis uses data collected in 2023 as part of two Dutch longitudinal surveys: the LISS panel (lissdata.nl) and the TRIAL panel. The LISS panel is a long-running study that implements all ethical guidelines to protect the privacy and integrity of its participants, in full compliance with Dutch and European legislation. Our module has undergone in-depth review by Centerdata (Tilburg University, the Netherlands), the data managing institution, to preclude questions that could, due to their content or wording, be perceived as threatening, offensive, or unpleasant by participants. It has additionally been reviewed by Centerdata's Data Protection Officer and Information Security Officer. Furthermore, the module complies with the Light Track framework for minimal-risk research established by the Social Sciences Ethics Committee of Radboud University Nijmegen, meeting essential ethical requirements regarding the recruitment of participants, their consent, rights and remuneration, risks and burdens imposed on participants, potential sensitivity of research materials, the absence of deception, and data protection. The TRIAL panel has been reviewed by the Ethics Committee Social Science at Radboud University Nijmegen (ECSW-LT-2022-11-30-15458). Participants of both surveys have given their explicit informed consent for their provided data to be processed for scientific purposes. We preregistered all experiments at the OSF (https://osf.io/xzq4k and https://osf.io/rdcez), and all replication materials are available at https://preference4similarity.netlify.app.

### Study design

Experiments 1 and 2 were fielded as part of the LISS panel, an Internet-based household survey administered by Centerdata at Tilburg University which maintains a representative sample of the Dutch population drawn and regularly refreshed from the administrative population register (78). Every month since 2007, LISS participants complete a questionnaire of annually recurring and additional one-off modules. Our experiments were part of such an additional module and administered to a subsample of 3,267 respondents who had indicated to be a member, participant, or volunteer in a civic organization in a LISS survey two months earlier and who had given their consent for their survey data to be linked to administrative register data. We specifically targeted civically active respondents to ensure that our subjects were familiar with making decisions about joining organizations and that the types of civic organizations shown to them are relevant to them. A total of 2,750 respondents, all aged 16 years or older, completed the module, for a response rate of 84.2%. Of these respondents, 2,733 and 2,743, respectively, had no missing responses for experiments 1 and 2.

In our module, respondents were subjected to two conjoint experiments with forced choices: one for neighborhoods and one for civic organizations. The order in which respondents completed the experiments was randomized, and each experiment was preceded by a question about how much respondents pay for their current housing/organization. This information was used to benchmark the financial costs that respondents would face in the conjoint experiments.

The introductory text for the experiments read: “Imagine you are leaving your current [neighborhood/organization] and are looking for a new [neighborhood/organization]. In the following questions, we each time give you two options. (…) Aside from practical features of the [neighborhoods/organizations], such as costs or vicinity, we also show information about the people who [live in these neighborhoods/are involved in these organizations]. Always pick the [neighborhood/organization] that suits you best.” We further clarified that neighborhoods concern all streets reachable within 5- to 10-min walking from respondents' new home and that organizations, in the case of larger federations, refer to the local branch the respondent would be involved in. The organizational profiles shown to respondents concerned the same type of organizations as they were involved in at the time (or spent most time on)—e.g. respondents involved in sports clubs had to choose between different sports club profiles. Finally, we clarified that the alternatives presented did not differ from one another apart from the characteristics shown on screen.

The neighborhood and organization profiles consisted of seven attributes: the three sociodemographic dimensions of interest (the shares of [neighbors/members] 50 years or older, with Turkish or Moroccan origins, and with a college degree) as well as several other relevant features of the alternatives (i.e. travel time to amenities/organization, average monthly costs, preexisting ties to [neighbors/members], and social cohesion). See Table S2 for the exact phrasing and potential values of all profile attributes. Based on these attributes and their potential values, there were 3,645 possible profiles per setting. Respondents were assigned four choice sets for each experiment, each consisting of two randomly sampled profiles (with replacement) from the respective profile universe, presented to respondents in tabular format. Moreover, we administered an additional iteration of the first choice set with a reversed profile order at the end of respondents' first experiment to estimate the reliability of their responses (see below). To prevent potential biases arising from the positioning of the attributes while limiting the cognitive load for respondents, we randomized the order of the attributes across but not within respondents. After completing the experiments, respondents answered further questions about their current neighborhoods and organizations, including about their composition along several social dimensions, which respondents could report using sliders (including a “don’t know” button). The responses to these questions were used to infer respondents' real-life exposure to in- and outgroups in the different settings. The full list of survey questions included in this data collection can be found at the end of the Supplement.

Experiment 3 was fielded as part of the third wave of the TRIAL panel, which is coordinated by a research consortium of the same name led by Radboud University Nijmegen (for more information, see 79). The TRIAL panel started in 2021 with a sample of 4,961 respondents aged 16 to 40 years randomly sampled from the Dutch population register by the survey agency I&O Research. The third survey wave was administered to 4,032 respondents (attrition rate until wave 3: 18.7%), of which 2,707 completed the module including experiment 3, for a response rate of 67.1%.

Experiment 3 concerned a similar forced-choice conjoint experiment as experiments 1 and 2, but this time focused on choices between paired profiles of sports clubs. The introductory text read: “In the following questions, we ask you to imagine you are looking for a new sports club, for example because you moved home. We are interested in what you find important about a new sports club. (…) We offer you three times a choice between two fictive sports clubs. You can assume the clubs are similar in all other respects. If you would actually rather not join either club, then choose the option that comes closest to your preferences.” Subsequently, the instructions for each choice task read: “Imagine you can choose between the following two sports clubs. Which one would you choose?” Similar to experiment 2, the profile attributes included travel time, preexisting ties to members, and the educational and ethnic composition of the club. In addition, we varied the composition of the team or training group the respondent would join. Note that ethnic composition was in this experiment measured as the percentage of members “with a migration background.” Finally, we varied the training frequency and the focus of the club (socially versus performance-oriented). See Table S6 for the exact phrasing and potential values of all profile attributes. Based on these attributes and their potential values, there were 5,400 possible profiles. Respondents were assigned three choice sets, each consisting of two profiles randomly sampled (with replacement) from the profile universe, presented to respondents in tabular format. Again, the order of the attributes was randomized across but not within respondents. The full list of survey questions included in this data collection can be found at the end of the Supplement.

### Statistical methods

To assess ingroup preferences across different sociodemographic subgroups, we calculated marginal means for each profile attribute, as marginal means are better suited for subgroup comparisons than other estimands commonly focused on in relation to conjoint experiments (66). Marginal means reflect the average probability with which a profile including a specific attribute level is chosen. In line with recommendations provided by Clayton and colleagues (65), we adjusted all our estimates for measurement error. To do so, we calculated subgroup-specific intrarespondent reliability scores for experiments 1 and 2. This was done based on a comparison between respondents' responses to their first choice set and a repetition of this choice set with the profile order reversed, which respondents were shown at the end of their first experiment. The reliability scores were used to estimate the amount of measurement error in respondents' response behavior, which we subsequently used to adjust the marginal means (for adjusting the marginal means from experiment 3 we used the measurement error estimates from experiment 2). We also formally tested whether the differences between subgroups' ingroup preferences in experiments 1 and 2 are statistically significant. For this purpose, we constructed, separately for each composition attribute and subgroup and based on 1,000 bootstrap iterations, a 95% CI around the difference in the conditional marginal means between the neighborhood and organization experiment. No survey weights were applied, and all analyses were carried out using RStudio version 4.4.0.

## Supplementary Material

pgaf256_Supplementary_Data

## Data Availability

In this study, we use data from the LISS and the TRIAL panel study. The LISS data can be accessed via https://www.dataarchive.lissdata.nl upon registration, which is free of charge. The relevant sections of the TRIAL data are available at the replication website for this study that also hosts all code needed to reproduce the results https://preference4similarity.netlify.app.
