# Supplementary material for: Ingroup preferences, segregation, and intergroup contact in neighborhoods and civic organizations
Source: PNAS Nexus. 2025 Sep 2;4(9):pgaf256. doi: 10.1093/pnasnexus/pgaf256 (PMC12403059; doi:10.1093/pnasnexus/pgaf256)
Supplement: pgaf256_Supplementary_Data [file pgaf256_supplementary_data.docx]

# Supplement to: Ingroup Preferences, Segregation, and Intergroup Contact in Neighborhoods and Civic Organizations

## Supplementary Materials Figure 2

Table S1: Descriptive overview of the sample underlying Experiments 1 and 2

|  | Mean | SD | Min | Max |
| --- | --- | --- | --- | --- |
| *Background characteristics* |  |  |  |  |
| Male | 0.51 | 0.50 | 0 | 1 |
| Female | 0.49 | 0.50 | 0 | 1 |
| Other / missing gender | 0.00 | 0.03 | 0 | 1 |
| Aged 50+ | 0.68 | 0.47 | 0 | 1 |
| Aged under 50 | 0.32 | 0.47 | 0 | 1 |
| College degree | 0.46 | 0.50 | 0 | 1 |
| No college degree | 0.52 | 0.50 | 0 | 1 |
| Education missing | 0.01 | 0.11 | 0 | 1 |
| Dutch background | 0.83 | 0.38 | 0 | 1 |
| Turkish / Moroccan background | 0.02 | 0.14 | 0 | 1 |
| Other migration background | 0.15 | 0.36 | 0 | 1 |
|  |  |  |  |  |
| *Most important organization* |  |  |  |  |
| Religious | 0.13 | 0.33 | 0 | 1 |
| Union | 0.06 | 0.23 | 0 | 1 |
| Sports | 0.45 | 0.50 | 0 | 1 |
| Environmental | 0.03 | 0.17 | 0 | 1 |
| Consumer | 0.06 | 0.23 | 0 | 1 |
| Cultural | 0.11 | 0.31 | 0 | 1 |
| Professional | 0.02 | 0.14 | 0 | 1 |
| Social | 0.04 | 0.20 | 0 | 1 |
| Political | 0.02 | 0.13 | 0 | 1 |
| Humanitarian | 0.02 | 0.14 | 0 | 1 |
| Educational | 0.02 | 0.14 | 0 | 1 |
| Migrants | 0.00 | 0.07 | 0 | 1 |
| Other type | 0.04 | 0.21 | 0 | 1 |
| Missing | 0.00 | 0.03 | 0 | 1 |
| N | 2,750 | | | |
| *Note: LISS panel. Supplemental table to Figure 2.* | | | | |

Table S2: Attributes and attribute levels (Experiments 1 and 2)

| Experiment 1: Choosing neighborhoods | Experiment 2: Choosing civic organizations |
| --- | --- |
| **Travel time to amenities (in minutes)**  10; 15; 20 | **Travel time from home (in minutes)**  10; 15; 20 |
| **Average monthly costs**  HomeCost*0.9; HomeCost *0.95; HomeCost *1; HomeCost *1.05; HomeCost *1.1 | **Average monthly costs**  OrgCost*0.7; OrgCost *0.9; OrgCost *1; OrgCost *1.1; OrgCost *1.3 |
| **Neighbors already known**  multiple; one; none | **Members already known**  multiple; one; none |
| **How well do most neighbors know each other**  by sight only; some by sight, others well; well | **How well do most members know each other**  by sight only; some by sight, others well; well |
| **Neighbors with college degree**  about a quarter (25%); about half (50%); about three quarters (75%) | **Members with college degree**  about a quarter (25%); about half (50%); about three quarters (75%) |
| **Neighbors with Turkish or Moroccan origins**  none (0%); a small minority (10%); about a quarter (25%) | **Members with Turkish or Moroccan origins**  none (0%); a small minority (10%); about a quarter (25%) |
| **Neighbors of 50 years or older**  about a quarter (25%); a little under half (40%); about half (50%) | **Members of 50 years or older**  about a quarter (25%); a little under half (40%); about half (75%) |

*Note: Attributes are displayed in bold. The profile universe of each conjoint experiment consists of 3x5x3x3x3x3x3 = 3,645 profiles. There are thus 6,641,190 possible choice sets, i.e., pairwise combinations of profiles, in each experiment. HomeCost refers to the amount of money respondents monthly spend on rent or mortgages for their current home. OrgCost refers to the financial contribution that respondents make to their current civic organization on a monthly basis (e.g., membership fees). Supplemental table to Figure 2.*

Figure S1: Marginal means for all attributes among the full sample (Experiments 1 and 2)


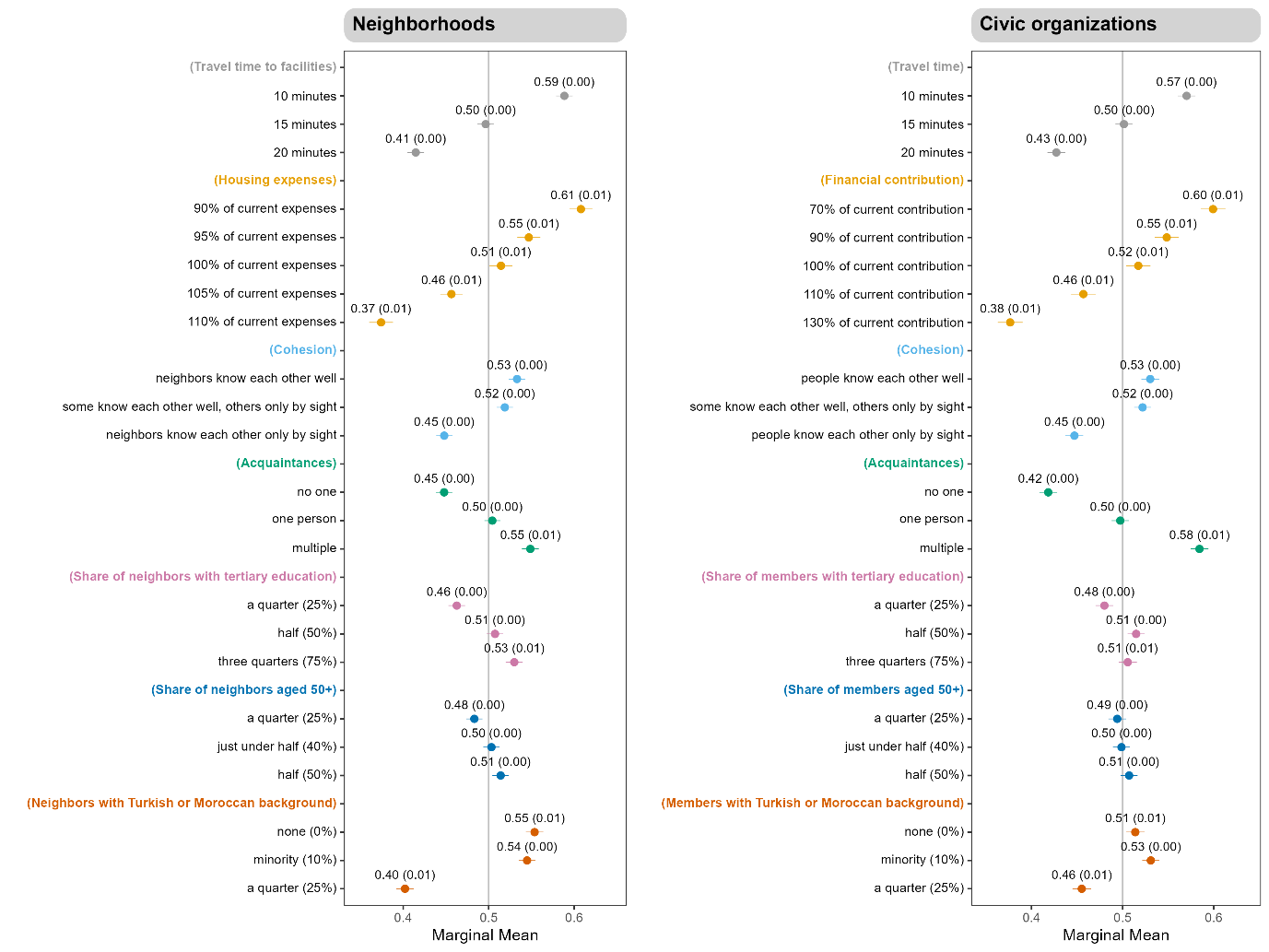


*Note: Marginal means reflect the average probability with which a profile with a particular attribute is chosen. Standard errors are displayed in parentheses and the error bars represent 95 percent confidence intervals. The instructions for each choice task read: “Imagine that you leave your current [neighborhood / civic organization] and are looking for a new one. Which one would you choose?” The civic organizations which respondents had to choose between were of the same type as their current (most time-intensive) organization. This figure is based on the choices made by 2,750 respondents, out of which 2,733 participated in the experiment on neighborhoods and 2,743 participated in the experiment on civic organizations. Each respondent was asked to complete four choice tasks for each setting. Supplemental figure to Figure 2.*

To formally test whether the subgroup-specific marginal means presented in Figure 2 differ between neighborhoods and civic organizations, additional regressions simultaneously including the data from Experiments 1 and 2 were run. In the last column of Table S3, we report bootstrapped 95% confidence intervals for the differences between the marginal means in the neighborhood vis-à-vis organizational context (Experiment 1 vis-à-vis Experiment 2). Confidence intervals excluding zero indicate that the difference between marginal means is statistically significant. Although this applies to nearly all of the confidence intervals in Table S3, for more than half of the comparisons the absolute value of the marginal mean difference is less than 0.02, highlighting that most subgroups still hold rather similar ingroup preferences regarding the composition of neighborhoods and civic organizations.

Table S3: Differences between neighborhood and organization choices across subgroups (Experiments 1 and 2)

| Respondent subgroup | Attribute level | MM Neighb. choice | MM Org. choice | Difference | Confidence interval around difference |
| --- | --- | --- | --- | --- | --- |
| Below 50 years | 25% 50 years or older | 0.535 | 0.572 | -0.038 | [-0.041, -0.039] |
| Below 50 years | 40% 50 years or older | 0.496 | 0.476 | 0.021 | [0.019, 0.021] |
| Below 50 years | 50% 50 years or older | 0.469 | 0.452 | 0.017 | [0.019, 0.021] |
| 50 years or older | 25% 50 years or older | 0.447 | 0.447 | 0.000 | [-0.001, 0.000] |
| 50 years or older | 40% 50 years or older | 0.509 | 0.510 | -0.002 | [-0.001, 0.001] |
| 50 years or older | 50% 50 years or older | 0.545 | 0.542 | 0.003 | [0.001, 0.003] |
|  |  |  |  |  |  |
| No migration background | 0% Turk./Moroc. background | 0.585 | 0.522 | 0.062 | [0.061, 0.063] |
| No migration background | 10% Turk./Moroc. background | 0.567 | 0.548 | 0.019 | [0.018, 0.019] |
| No migration background | 25% Turk./Moroc. background | 0.351 | 0.429 | -0.078 | [-0.079, -0.077] |
| Turk./Moroc. background | 0% Turk./Moroc. background | 0.341 | 0.000 | 0.341 | * |
| Turk./Moroc. background | 10% Turk./Moroc. background | 0.575 | 0.638 | -0.062 | * |
| Turk./Moroc. background | 25% Turk./Moroc. background | 0.581 | 0.910 | -0.329 | * |
|  |  |  |  |  |  |
| Without college degree | 25% with college degree | 0.486 | 0.515 | -0.029 | [-0.031, -0.029] |
| Without college degree | 50% with college degree | 0.507 | 0.515 | -0.008 | [-0.008, -0.006] |
| Without college degree | 75% with college degree | 0.506 | 0.469 | 0.037 | [0.037, 0.038] |
| With college degree | 25% with college degree | 0.410 | 0.426 | -0.017 | [-0.018, -0.016] |
| With college degree | 50% with college degree | 0.513 | 0.525 | -0.012 | [-0.014, -0.012] |
| With college degree | 75% with college degree | 0.579 | 0.551 | 0.028 | [0.029, 0.031] |

*Note: MM=Marginal mean. Turk.=Turkish, Moroc.=Moroccan. Subtracting the marginal means in the “Organization choice” column from those in the “Neighborhood choice” column yields the values in the “Difference” column. The confidence intervals are approximated based on 1,000 bootstrap iterations. Because the number of respondents with a Turkish or Moroccan background is comparatively small, no reliable confidence intervals can be produced for these subgroups. Supplemental table to Figure 2.*

Table S4 reports the differences in the strength of preferences revealed in Experiments 1 and 2 for each subgroup and each attribute. Combining information from Table S3, column 3 reports for each subgroup by how much the marginal means change when moving from the first composition attribute (e.g., 25% 50 years or older: 0.534) to the second (e.g., 40% 50 years or older: 0.498). Column 4 reports by how much the marginal means of a choice option decline among a subgroup for each additional minute of travel time. To obtain these figures, the marginal mean of a choice option involving 20 minutes travel time is subtracted from the marginal mean of a choice option involving 10 minutes travel time and divided by 10. This calculation is apposite because we find almost perfect linearity in how individuals respond to the travel time attributes. That is, the marginal means for choice options with 15 minutes travel time lie exactly in the middle between those with 10 and 20 minutes travel time. Column 5 reports the ratio of the values in columns 3 and 4 multiplied by -1, indicating the change in travel time to which the social composition change is equivalent. The values in column 5 can therefore be interpreted as the amount of additional time that members of a given subgroup are willing to travel to attain a setting with the latter vis-à-vis the former social composition. For example, those above 50 years are willing to accept 3.896 minutes more travel time to key amenities if their new neighborhood is made up of 50 rather than 25 percent residents above 50 years. Conversely, those below 50 years would require 2.933 minutes less travel time to key amenities in the older neighborhood to be indifferent between neighborhoods with these age compositions.

Table S4: Benchmarking ingroup preferences against preferences for reduced travel time (Experiments 1 and 2)

| Setting (1) | Focal subgroup  (2) | Difference in MM when switching from one social composition to another  (3) | Decrease in MM associated with one additional minute of travel time  (4) | Exchangeable travel time for switch in social composition  [in min]  (5) |
| --- | --- | --- | --- | --- |
| Neighborhood | Below 50 years | 25 🡪 40% 50 years or older: -0.039 | -0.022 | -1.741 |
|  | Below 50 years | 40 🡪 50% 50 years or older: -0.027 | -0.022 | -1.205 |
|  | Below 50 years | 25 🡪 50% 50 years or older: -0.066 | -0.022 | -2.946 |
|  | 50 years or older | 25 🡪 40% 50 years or older: 0.062 | -0.025 | 2.480 |
|  | 50 years or older | 40 🡪 50% 50 years or older: 0.036 | -0.025 | 1.440 |
|  | 50 years or older | 25 🡪 50% 50 years or older: 0.098 | -0.025 | 3.920 |
|  | No migration backgr. | 0 🡪 10% Turk. or Moroc. backgr.: -0.018 | -0.024 | -0.756 |
|  | No migration backgr. | 10 🡪 25% Turk. or Moroc. backgr.: -0.216 | -0.024 | -9.076 |
|  | No migration backgr. | 0 🡪 25% Turk. or Moroc. backgr.: -0.234 | -0.024 | -9.832 |
|  | Turk. or Moroc. backgr. | 0 🡪 10% Turk. or Moroc. backgr.: 0.234 | -0.015 | 15.395 |
|  | Turk. or Moroc. backgr. | 10 🡪 25% Turk. or Moroc. backgr.: 0.006 | -0.015 | 0.395 |
|  | Turk. or Moroc. backgr. | 0 🡪 25% Turk. or Moroc. backgr.: 0.240 | -0.015 | 15.789 |
|  | Without college degree | 25 🡪 50% With college degree: 0.021 | -0.026 | 0.981 |
|  | Without college degree | 50 🡪 75% With college degree: -0.001 | -0.026 | -0.047 |
|  | Without college degree | 25 🡪 75% With college degree: 0.020 | -0.026 | 0.935 |
|  | With college degree | 25 🡪 50% With college degree: 0.103 | -0.021 | 3.902 |
|  | With college degree | 50 🡪 75% With college degree: 0.066 | -0.021 | 2.500 |
|  | With college degree | 25 🡪 75% With college degree: 0.169 | -0.021 | 6.402 |
| Organizations | Below 50 years | 25 🡪 40% 50 years or older: -0.096 | -0.024 | -3.934 |
|  | Below 50 years | 40 🡪 50% 50 years or older: -0.024 | -0.024 | -0.984 |
|  | Below 50 years | 25 🡪 50% 50 years or older: -0.120 | -0.024 | -4.918 |
|  | 50 years or older | 25 🡪 40% 50 years or older: 0.063 | -0.017 | 3.621 |
|  | 50 years or older | 40 🡪 50% 50 years or older: 0.032 | -0.017 | 1.839 |
|  | 50 years or older | 25 🡪 50% 50 years or older: 0.095 | -0.017 | 5.460 |
|  | No migration backgr. | 0 🡪 10% Turk. or Moroc. backgr.: 0.026 | -0.020 | 1.313 |
|  | No migration backgr. | 10 🡪 25% Turk. or Moroc. backgr.: -0.119 | -0.020 | -6.010 |
|  | No migration backgr. | 0 🡪 25% Turk. or Moroc. backgr.: -0.093 | -0.020 | -4.697 |
|  | Turk. or Moroc. backgr. | 0 🡪 10% Turk. or Moroc. backgr.: 0.638 | -0.017 | 38.667 |
|  | Turk. or Moroc. backgr. | 10 🡪 25% Turk. or Moroc. backgr.: 0.272 | -0.017 | 16.485 |
|  | Turk. or Moroc. backgr. | 0 🡪 25% Turk. or Moroc. backgr.: 0.910 | -0.017 | 55.152 |
|  | Without college degree | 25 🡪 50% With college degree: 0.000 | -0.020 | -0.000 |
|  | Without college degree | 50 🡪 75% With college degree: -0.046 | -0.020 | -2.371 |
|  | Without college degree | 25 🡪 75% With college degree: -0.046 | -0.020 | -2.371 |
|  | With college degree | 25 🡪 50% With college degree: 0.099 | -0.019 | 4.853 |
|  | With college degree | 50 🡪 75% With college degree: 0.026 | -0.019 | 1.275 |
|  | With college degree | 25 🡪 75% With college degree: 0.125 | -0.019 | 6.127 |

*Note: Supplemental table to Figure 2.*

Figure S2 replicates Figure 2 in the main text using only data from the first experiment that each respondent completed, to rule out any bias that may result from the same respondents participating in both experiments. About one half of the respondents first completed the neighborhood experiment (N=1,355), the other half the organization experiment (N=1,406). Each respondent completed four choice tasks per experiment.

The results displayed in this figure support the view that the similarity of ingroup preferences observed across the two settings is not driven by respondents developing response heuristics in the first experiment and subsequently applying these heuristics mindlessly in the second experiment. Likewise, restricting the analyses summarized in Figure 3 in the main text to the first experiment each respondent completed does not result in meaningful changes (not shown in this supplement).

Figure S2: Replicating Figure 2 exclusively with data from the first experiment completed by each respondent (Experiments 1 and 2)


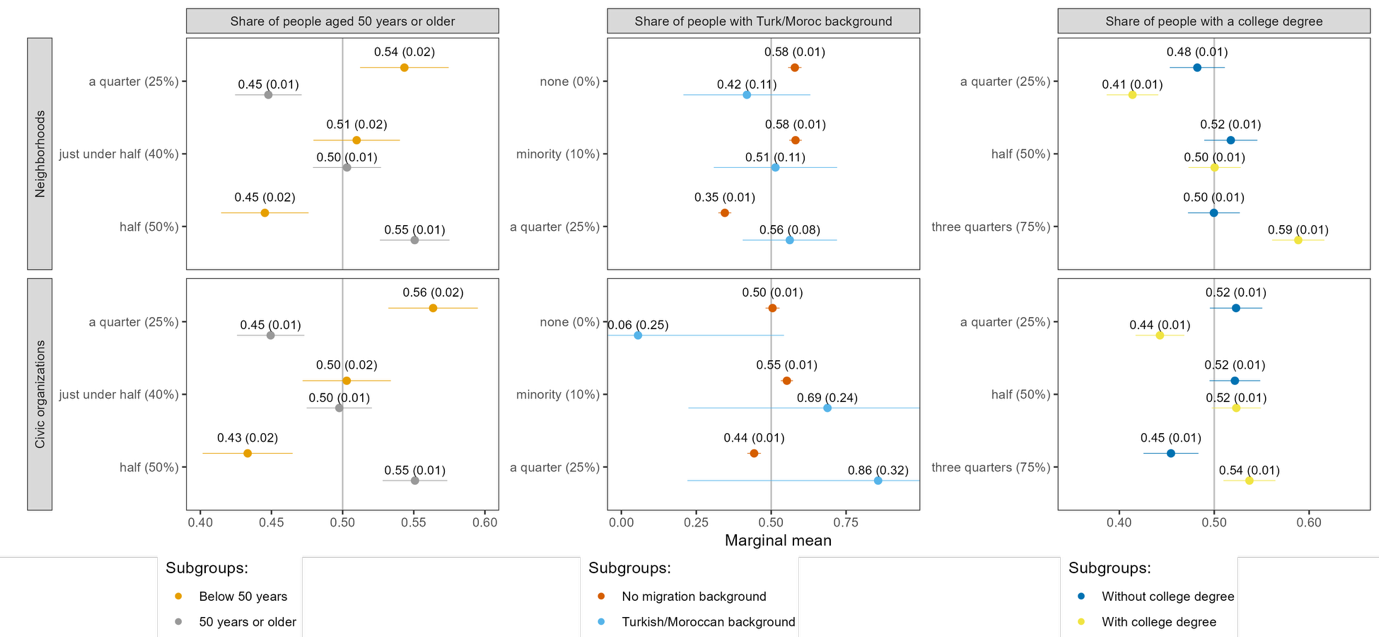


*Note: Marginal means reflect the average probability with which a profile with a particular attribute is chosen. Standard errors are displayed in parentheses and the error bars represent 95 percent confidence intervals. All estimates have been corrected for measurement error following Clayton et al. (2023)^1^. The instructions for each choice task read: “Imagine you leave your current [neighborhood / civic organization] and are looking for a new one. Which one would you choose?” The civic organizations which respondents had to choose between were of the same type as their current (most time-consuming) organization. This figure is based on the choices made by 2,750 respondents, out of which 1,355 participated in Experiment 1 first and out of which 1,406 participated in Experiment 2 first. Each respondent was asked to complete four choice tasks for each setting but only responses to the first experiment they were exposed to are included in this figure. Supplemental table to Figure 2.*

## Supplementary Materials Figure 3

Figure S3: Marginal means of neighborhoods’ and organizations’ social composition attributes by the composition of respondents’ current settings, for all subgroups (Experiments 1 and 2)


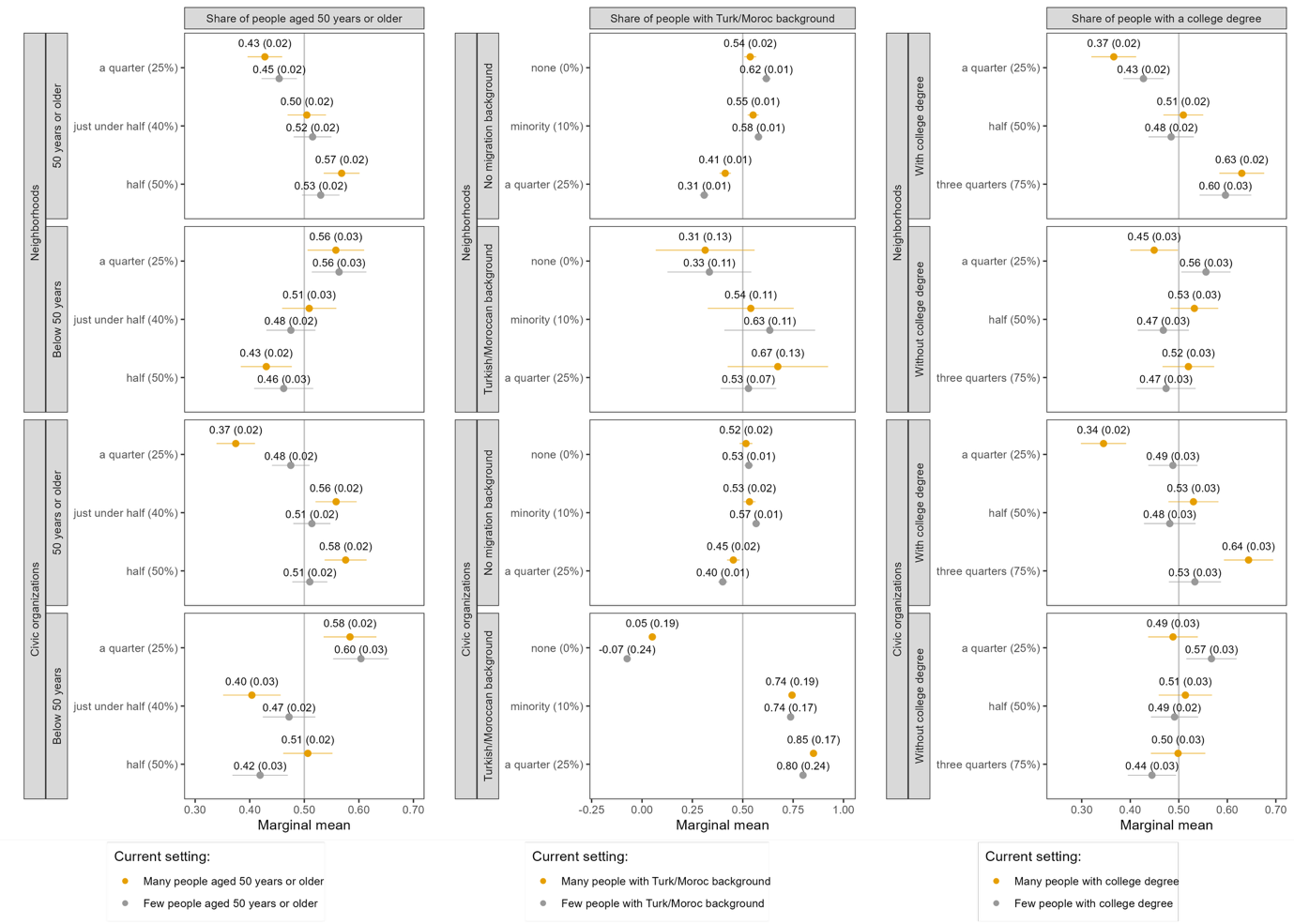


*Note: Marginal means reflect the average probability with which a profile with a particular attribute is chosen. Standard errors are displayed in parentheses and the error bars represent 95 percent confidence intervals. All estimates have been corrected for measurement error following Clayton et al. (2023) ^1^. Confidence intervals for organizational estimates of respondents with a Turkish or Moroccan background were not displayed because they extended beyond the x-axis range. The instructions for each choice task read: “Imagine that you leave your current [neighborhood / civic organization] and are looking for a new one. Which one would you choose?” The civic organizations respondents had to choose between were of the same type as their current (most time-intensive) organization (e.g., different sports clubs). The composition of respondents’ current neighborhoods and organizations was estimated by the respondents; see the notes to Figure 1 in the main text for the wording of the associated survey questions. Respondents who reported not to know the composition of their current settings have been excluded. Settings with “many” [“few”] people aged 50 years or older / with a college degree refer to those in the highest [lowest] quartile in the distribution of exposure to people with these characteristics. Settings with “many” [“few”] people with a Turkish/Moroccan background refer to those above [below] the mean share of residents with a Turkish/Moroccan background. This figure is based on a smaller number of respondents than Figure 2 (N=2,588 instead of 2,750) because respondents who reported not to know the composition of their setting were excluded. Respondents were asked to complete four choice sets per setting. Supplemental figure to Figure 3.*

In Figure S4, we replicate the neighborhood panels of Figure 3. In this figure, we define respondent subgroups with ‘many’ and ‘few’ people with a certain characteristic in their current neighborhood based on administrative records of neighborhood composition instead of respondents’ reports through the survey. The patterns observed in Figure S4 largely mirror those presented in Figure 3. In accordance with Figure 3, Figure S4 shows for ethnicity and education that ingroup preferences are stronger among individuals who are exposed to fewer outgroup members in their current neighborhood. For age, Figure S4 suggests that ingroup preferences are somewhat more pronounced among respondents aged 50 or older living in neighborhoods with many vis-à-vis few people aged 45 or older. However, these differences are statistically insignificant.

Figure S4: Replicating Figure 3 using administrative records of neighborhood composition (Experiment 1)


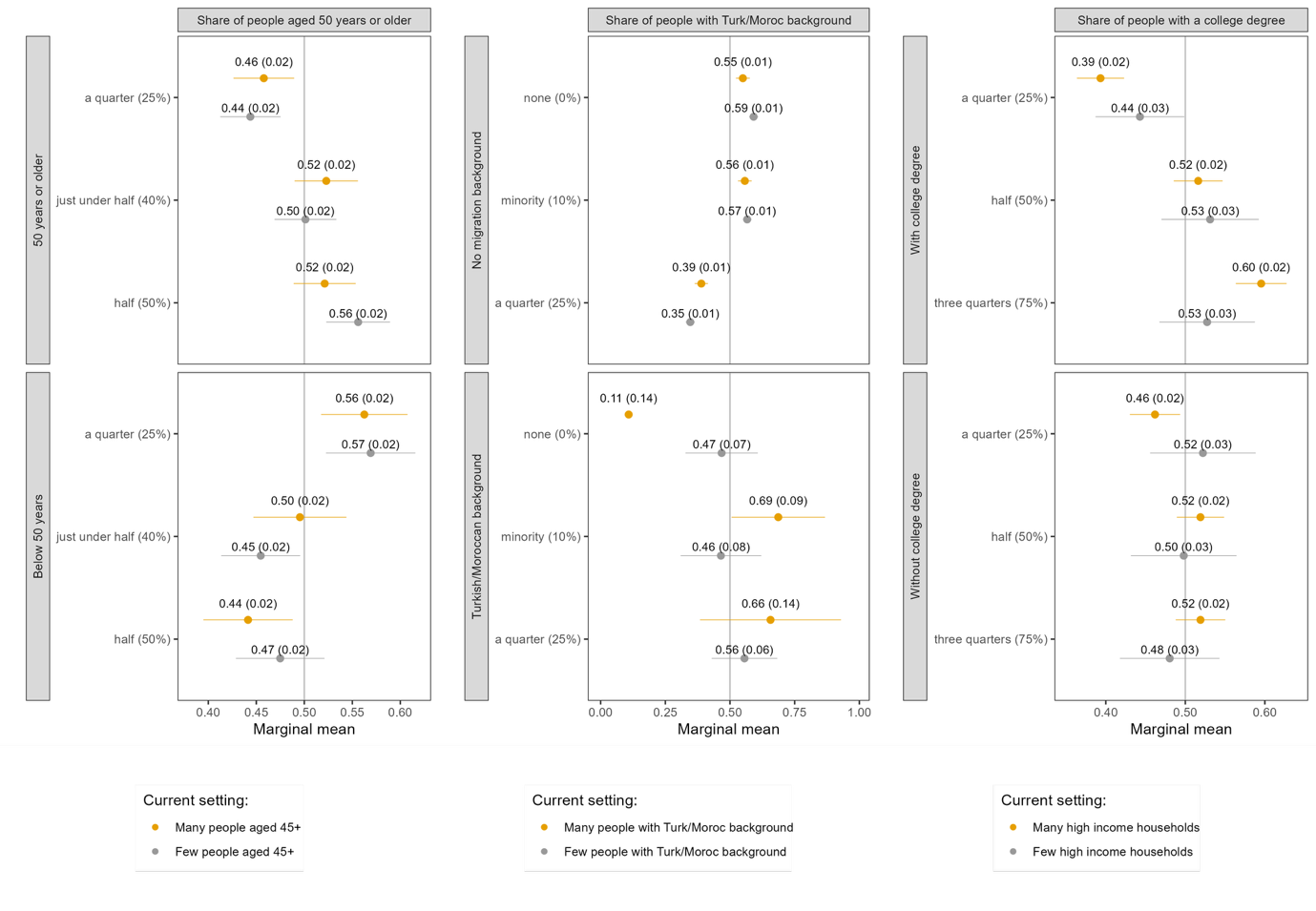
*Note: Marginal means reflect the average probability with which a profile with a particular attribute is chosen. Standard errors are displayed in parentheses and the error bars represent 95 percent confidence intervals. All estimates have been corrected for measurement error following Clayton et al. (2023) ^1^. The instructions for each choice task read: “Imagine that you leave your current neighborhood and are looking for a new one. Which one would you choose?”. Current settings with “many” [“few”] people aged 45 or older refer to neighborhoods in the highest [lowest] quartile in the distribution of exposure to people aged 45 or older. Current settings with “many” [“few”] people with a Turkish/Moroccan background refer to those above [below] the mean share of residents with a Turkish/Moroccan background. Current settings with “many” [“few”] high income households refer to neighborhoods in the highest [lowest] quartile of exposure to households with an income exceeding those of 80% of households in the Netherlands. All information on respondents’ current neighborhoods is derived from the administrative registers held by Statistics Netherlands. Note that a neighborhood’s share of high-income households is used as a proxy for the presence of neighbors with a college degree, for which neighborhood-level data is not available. For similar reasons, we consider here neighborhoods’ shares of residents aged over 45 to measure their age composition. This figure is based on the choices made by 2,700 respondents. Each respondent was asked to complete four choice tasks for each setting. Supplemental figure to Figure 3.*

## Supplementary Materials Figure 4

Table S5: Descriptive overview of the sample underlying Experiment 3

|  | Mean | SD | Min | Max |
| --- | --- | --- | --- | --- |
| Male | 0.33 | 0.47 | 0 | 1 |
| Female | 0.66 | 0.47 | 0 | 1 |
| Other / missing gender | 0.01 | 0.07 | 0 | 1 |
| Age | 31.37 | 56.09 | 18 | 41 |
| College degree | 0.76 | 0.42 | 0 | 1 |
| No college degree | 0.24 | 0.42 | 0 | 1 |
| Dutch background | 0.89 | 0.31 | 0 | 1 |
| Migration background | 0.11 | 0.31 | 0 | 1 |
| N | 2,707 | | | |

*Note: Wave 3 of the TRIAL survey. Supplemental table to Figure 4.*

Table S6: Attributes and attribute levels (Experiment 3)

| Experiment 3: Choosing sports clubs |
| --- |
| **Travel time from home (in minutes)**  5; 15; 30 |
| **Training frequency**  once a week; more than once a week |
| **Members already known**  none; multiple |
| **Club ethos as indicated by a statement on the club’s website**  exercising together is building friendships; lift yourself to a higher level |
| **Share of club members with a college degree**  about a quarter (25%); about half (50%); about three quarters (75%) |
| **Share of training group members with a college degree**  a small minority (10%); about a quarter (25%); about half (50%); about three quarters (75%); a large majority (90%) |
| **Share of club members with a migration background**  a few (5%); a small minority (10%); about a quarter (25%) |
| **Share of training group members with a migration background**  none (0%); a few (5%); a small minority (10%); about a quarter (25%); about half (50%) |

*Note: Attributes are displayed in bold. The profile universe of the conjoint experiment consists of 3x2x2x2x3x5x3x5 = 5,400 profiles. There are thus 14,577,300 possible choice sets, i.e., pairwise combinations of profiles. Supplemental table to Figure 4.*

Figure S5: Marginal means for all attributes among the full sample (Experiment 3)


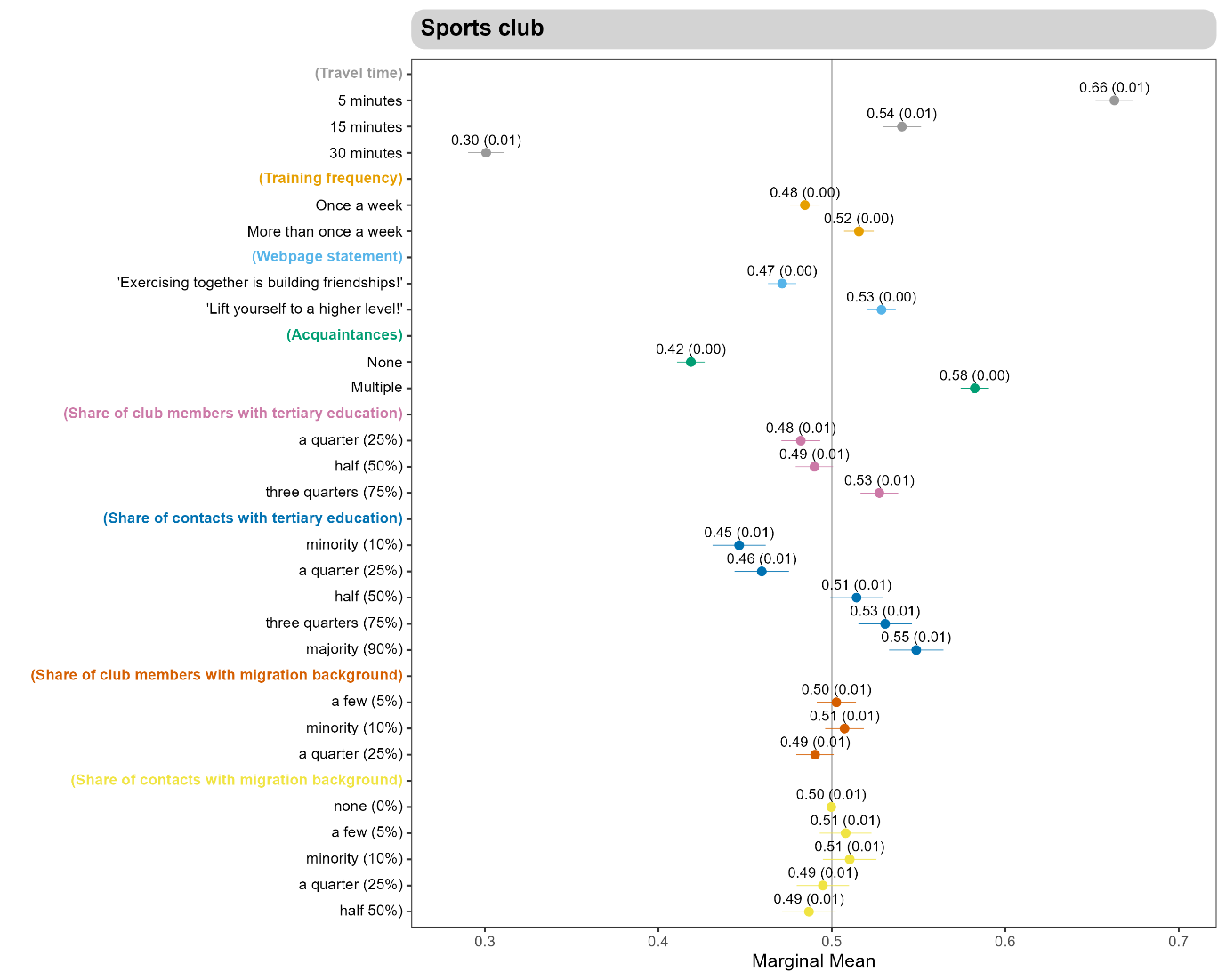


*Note: Marginal means reflect the average probability with which a profile with a particular attribute is chosen. Standard errors are displayed in parentheses and the error bars represent 95 percent confidence intervals. The instructions for the choice task read: “Imagine that you have to choose between the following sports clubs. Which one would you choose?”. This figure is based on the choices made by 2,707 respondents. Each respondent was asked to complete three choice tasks. Supplemental figure to Figure 4.*

Figure S6: Re-analyzing Experiment 2 by type of organization and applying the age restrictions from Experiment 3 (only respondents aged up to 40)

*
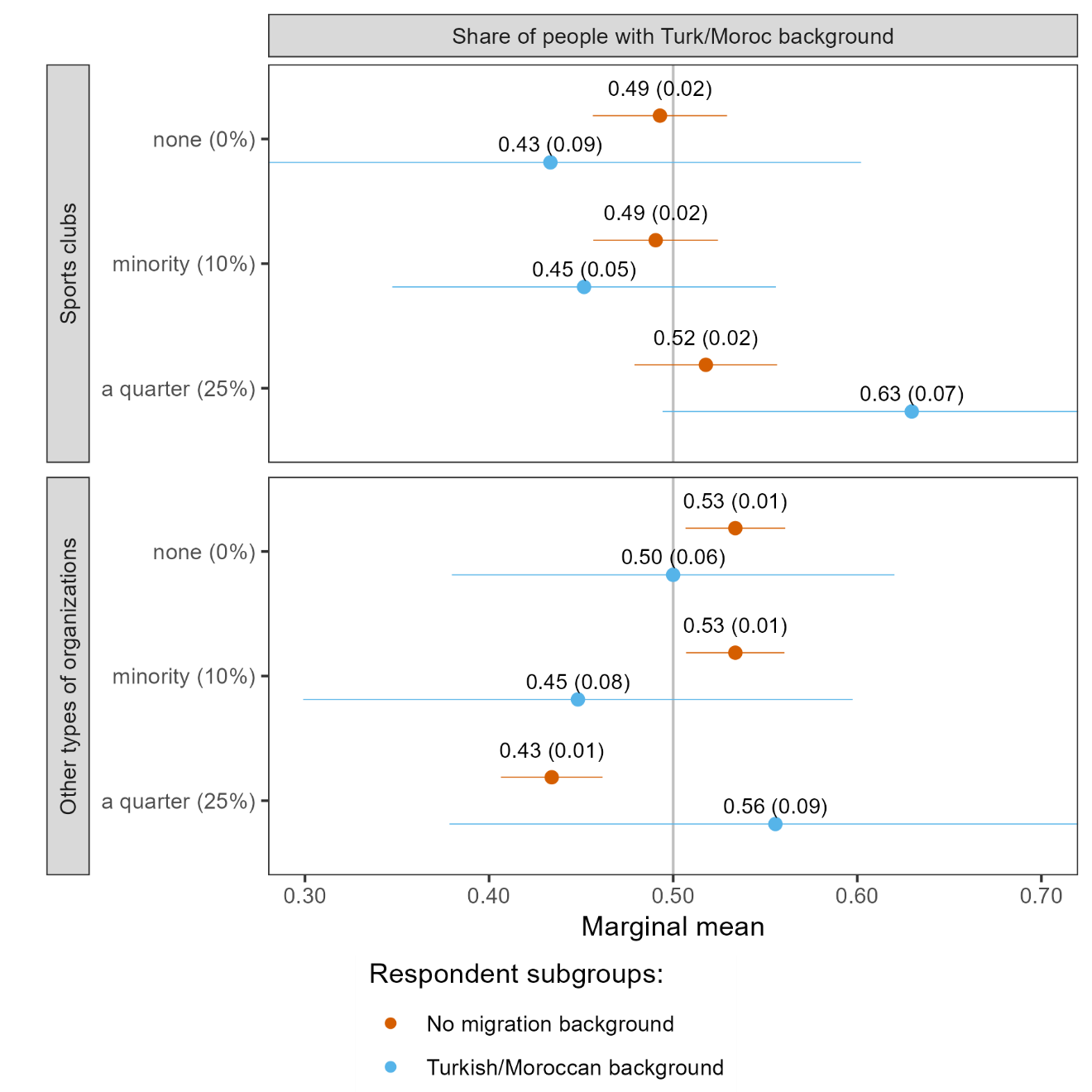
*

*Note: Marginal means reflect the average probability with which a profile including a particular attribute is chosen. Standard errors are displayed in parentheses and the error bars represent 95 percent confidence intervals. The instructions for each choice task read: “Imagine you leave your current civic organization and are looking for a new one. Which one would you choose?” The civic organizations which respondents had to choose between were of the same type as their current (most time-consuming) organization. Restricted to respondents aged 40 or under (the same age range as the sample underlying Experiment 3). This figure is based on the choices made by 604 respondents. Each respondent was asked to complete four choice tasks. Supplemental figure to Figure 4.*

## Questionnaire – Experiments 1 and 2

Random_group_variable = Variable that randomly assigns respondents to two groups (0 / 1)

This questionnaire is about associations and neighborhoods.

**Q01** You recently indicated that you were involved with the following organization or organizations as a member, volunteer or participant.

Please indicate which of these organizations you are still involved in as a member, volunteer or participant.

Sports club

Cultural association or hobby club

Labor union

Business, professional or agricultural organization

Consumers’ organization or automobile association

Organization for humanitarian aid or human rights

Organization for migrants

Environmental protection organization, peace organization or animal rights organization

Religious or church organization

Political party

Science, education, teachers’ or parents’ association

Members’ club / association for youth, pensioners/senior citizens, women, societies of friends

Other organization that allows people to join voluntarily

I am no longer involved with any of these organizations.

(*Pre-load organizations for which the respondent was a member, a volunteer, or a participant in Wave 16 of the Social Integration & Leisure module, Oct/Nov 2023. If the respondent is no longer involved in any of these organizations, the rest of Part 1 will be skipped.*)

**Q02** (*if the number of selected organizations in Q01 > 1*) Which organization do you spend the most time on? */ if respondent is no longer involved in any organization:* You indicated that you are no longer involved with the following organization or organizations. When you were still involved, which organization did you spend the most time on?

(*Display only those organizations the respondent is still involved in according to Q01, store response as ‘Org1’.*)

**Q03** What was the total amount that you paid to your **[Org1]** every month? / *else:* What is the total amount that you pay to your **[Org1]** every month?]

*If you do not know exactly, you may provide an estimate.*

___ Euro

I don’t know

I don’t want to say it

(Pre-load [Org1] from Q01/Q02 for this and all following questions in Part 1)

**Introduction to Q04** [*if Q01=1*: You indicated that you are no longer involved with the previously mentioned [Org1]. Imagine that you were to look for a new [Org1]. / *else:* Imagine that you were to leave your current [Org1] and look for a new [Org1].]

In each of the following [*if random_group_variable = 0:* four /*if random_group_variable = 1:* five] questions, we will show you two options. These options concern the details of the local divisions of these organizations. Aside from practical aspects, such as cost and accessibility, this will also include information about the people involved with these organizations.

In each question, please choose the [Org1] that suits you best.

**Q04a-d/e** (if random_group_variable = 0, then Q04a-d; if random_group_variable = 1, then Q04a-e)

If you are using a smartphone to complete the questionnaire, please hold your phone horizontally for this question.

Imagine that you were to look for a new [Org1]. / *else:* Imagine that you were to leave your current [Org1] and look for a new [Org1].]

Which organization would you choose?

|  | Organization 1 | Organization 2 |
| --- | --- | --- |
| Dimension 1 | Value | Value |
| Dimension 2 | Value | Value |
| Dimension 3 | Value | Value |
| Dimension 4 | Value | Value |
| Dimension 5 | Value | Value |
| Dimension 6 | Value | Value |
| Dimension 7 | Value | Value |

The organizations are identical in all other respects.

Organization 1

Organization 2

*In the organization choice experiment, the dimensions were shown in a random order.*

**Introduction to Q05-Q10** We will now ask a few questions about the people at your *[***former/current**] [Org1].

If your [former /current] [Org1] is a local division of a larger (e.g. national) organization, please answer these questions with regard to the local division where you [were/are] active.

If you [were/are] involved with multiple [Org1], please answer the questions with regard to the organization you [spent/spend] the greatest amount of time on.

**Q05** How often [did / do] you attend gatherings or participate in activities organized by your [former/current] [Org1]?

Less than once per month

One to three times per month

Once per week

More than once per week

Don’t know

**Q06** Approximately how many people are involved with your [former/current] [Org1]?

*If you do not know exactly, you may estimate the number. If your [former/current] [Org1] is a local branch of a larger (national) organization, please answer the questions for the local chapter in which [ was/are] active.*

*Fewer than 20 people*

*20 to 49 people*

*50 to 99 people*

*100 to 199 people*

*200 to 499 people*

*500 or more people*

**Q07** How many people in your [former/current] [Org1] [did/do] you regularly interact with?

*These are people you know by name or regularly [talk/talked] with within the organization. If you do not know exactly, you may estimate the number.*

*Nobody*

*1 to 4*

*5 to 9*

*10 to 19*

*20 to 49*

*50 or more*

**Q08** How [did/do] you like it at your [former/current] [Org1]?

Slider: Not at all (0) – Very much (100)

**Q09** Now please think about **everyone who is part** of your [*former*/current] [Org1] (e.g. members and volunteers).

These do not have to be people who you know by name or [interacted/interact] with. If your [*former*/ current] [Org1] is a local division of a larger organization, we would again like you to base your answer on the local division within which you [were/are] active.

What percentage of these people...

*Please try to be as accurate as possible. If you do not know exactly, you may provide an estimate. If you really have no idea, then you can say so.*

*… are women? (Slider 0-100%, checkbox: I really don’t know)*

*… are 50 years or older? (Slider 0-100%, checkbox: I really don’t know)*

*… have a Turkish or Moroccan background? (Slider 0-100%, checkbox: I really don’t know)*

*… have* a higher professional education (Dutch: *HBO*) or university degree? *(Slider 0-100%, checkbox: I really don’t know)*

**Q10** Now please think about **the** [response to Q07] **people you regularly** [**interacted/interact**] **with**.

If your [former/current] [Org1] is a local division of a larger organization, we would again like you to base your answer on the local division within which you [were/are] active.

What percentage of these people...

*Please try to be as accurate as possible. If you do not know exactly, you may provide an estimate. If you really have no idea, then you can say so.*

*… are women? (Slider 0-100%, checkbox: I really don’t know)*

*… are 50 years or older? (Slider 0-100%, checkbox: I really don’t know)*

*… have a Turkish or Moroccan background? (Slider 0-100%, checkbox: I really don’t know)*

*… have* a higher professional education (Dutch: *HBO*) or university degree? *(Slider 0-100%, checkbox: I really don’t know)*

**Q11** We will now ask a few questions about your housing situation.

Please indicate how much your household pays in housing expenses (net) per month, approximately.

*Include all expenses related to your home, including rent or mortgage payments, gas, water and power, service and Homeowners’ Association fees. If you do not know exactly, you may provide an estimate.*

___ Euro

**Introduction to Q12** Imagine that you were to leave your current neighborhood and look for a new neighborhood to live in.

In the following [*if random_group_variable = 0:* 5 / *else:* 4] questions, you will be shown two options each time. The characteristics listed for each neighborhood apply to all the streets within a 5 to 10-minute walk from your new home. Aside from practical details, such as housing costs and the accessibility of amenities, you will also see information about the people who live in these neighborhoods.

Please choose the neighborhood that would suit you best.

**Q12a-d/e** (if random_group_variable = 0, then Q04a-e; if random_group_variable = 1, then Q04a-d)

If you are using a smartphone to complete the questionnaire, please hold your phone horizontally for this question.

Imagine that you were to leave your current neighborhood and look for a new neighborhood to live in.

Which neighborhood would you choose?

|  | Neighborhood 1 | Neighborhood 2 |
| --- | --- | --- |
| Dimension 1 | Value | Value |
| Dimension 2 | Value | Value |
| Dimension 3 | Value | Value |
| Dimension 4 | Value | Value |
| Dimension 5 | Value | Value |
| Dimension 6 | Value | Value |
| Dimension 7 | Value | Value |

The neighborhoods are identical in all other respects.

Neighborhood 1

Neighborhood 2

*In the neighborhood choice experiment, the dimensions were shown in a random order.*

**Introduction Q13-16** Finally, we would like to ask a few questions about your **current** neighborhood.

By your neighborhood, we mean all streets within a 5 to 10-minute walk from your home.

**Q13** Approximately how many people in your current neighborhood do you have regular contact with? (Do not count the people in your household)

*These are people you know by name or regularly talk with. If you do not know exactly, you may estimate the number.*

Nobody

1 to 4

5 to 9

10 to 19

20 to 49

50 or more

**Q14** How do you like your current neighborhood?

Slider Not at all (0) – Very much (100)

**Q15** Now please think about **all the people who live in your neighborhood**.

These do not need to be people you know by name or have contact with. By your neighborhood, we mean all streets within a 5 to 10-minute walk from your home.

What percentage of these people...

*Please try to be as accurate as possible. If you do not know exactly, you may provide an estimate. If you really have no idea, then you can say so.*

… are women? (Slider 0-100%, checkbox: I really don’t know)

… are 50 years or older? (Slider 0-100%, checkbox: I really don’t know)

… have a Turkish or Moroccan background? (Slider 0-100%, checkbox: I really don’t know)

… have a higher professional education (Dutch: HBO) or university degree? (Slider 0-100%, checkbox: I really don’t know)

**Q16** Now please think of **the** [**response to Q13**] **people you have regular contact with** within your current neighborhood.

By your neighborhood, we mean all streets within a 5 to 10-minute walk from your home.

What percentage of these people...

*Please try to be as accurate as possible. If you do not know exactly, you may provide an estimate. If you really have no idea, then you can say so.*

… are women? (Slider 0-100%, checkbox: I really don’t know)

… are 50 years or older? (Slider 0-100%, checkbox: I really don’t know)

… have a Turkish or Moroccan background? (Slider 0-100%, checkbox: I really don’t know)

… have a higher professional education (Dutch: HBO) or university degree? (Slider 0-100%, checkbox: I really don’t know)

The full questionnaires of all waves of the LISS survey can be found in the LISS archive (<https://www.lissdata.nl/how-it-works-archive>)

## Questionnaire – Experiment 3

**Introduction Q01** In the following questions, we are going to ask you to imagine that you are looking for a new sports club, for example because you have moved houses.

We are interested in what you deem important when you choose a new sport club. How important are commuting time, the training schedule and the diversity of the sport club?

Do you prefer a club with many members with a migration background (e.g., of Moroccan, Polish, or Surinamese background) or not? Is members’ education level important?

We are not only looking at the composition of the entire club, but also at the composition of your training group – the club members you will train together with.

There will be three choices between two fictional sports clubs each. Assume that these options are identical in all other respects. In case you do not want to join any of the sports clubs/centers, please pick to option that comes closest to your preferences.

**Q01-03** Imagine that you can choose between the following two options. Please pick the sports club would you choose?

|  | Sports club/center 1 | Sports club/center 2 |
| --- | --- | --- |
| Dimension 1 | Value | Value |
| Dimension 2 | Value | Value |
| Dimension 3 | Value | Value |
| Dimension 4 | Value | Value |
| Dimension 5 | Value | Value |
| Dimension 6 | Value | Value |
| Dimension 7 | Value | Value |
| Dimension 8 | Value | Value |

Sports club/center 1

Sports club/center 2

**Q04** What is the percentage of people with a migration background (e.g., Moroccan, Polish, Surinamese) among …

1. The members of your sports club/center. (slider 5 percentage point steps)
2. The members of your sports club/center with whom you have a lot of contact (e.g., in your team or training group)? (slider 5 percentage point steps)
3. The inhabitants of your neighborhood? (slider 5 percentage point steps)
4. The inhabitants of your street? (slider 5 percentage point steps)

**Q05** What is the percentage of people who completed a college degree or are studying towards this among …

1. The members of your sports club/center. (slider 5 percentage point steps)
2. The members of your sports club/center with whom you have a lot of contact (e.g., in your team or training group)? (slider 5 percentage point steps)
3. The inhabitants of your neighborhood? (slider 5 percentage point steps)
4. The inhabitants of your street? (slider 5 percentage point steps)

**Q06** We would like to know what you think about different groups in the Netherlands. We ask about this using a thermometer score. A score between 50 and 100 degrees corresponds to positive or warm feelings towards a group. A score between 0 and 50 degrees corresponds to negative or cold feelings towards a group. A score of 50 corresponds to neutral feelings.

1. People of Dutch background (thermometer slider 0-100 [cold-warm])
2. People with a migration background (e.g., Moroccan, Polish, Surinamese) (thermometer slider 0-100 [cold-warm])
3. People with a college degree (thermometer slider 0-100 [cold-warm])
4. People without a college degree (thermometer slider 0-100 [cold-warm])

## References

1. Clayton, Katherine, Yusaku Horiuchi, Aaron Kaufman, Gary King, and Mayya Komisarchik. 2023. “Correcting Measurement Error Bias in Conjoint Survey Experiments.” *American Journal of Political Science* (12):1–11.
